# Negligence in biomedical research: an anti-racist approach for substance use researchers

**DOI:** 10.3389/fpubh.2024.1401221

**Published:** 2024-07-31

**Authors:** Jonathan Lehman, Danniella Balangoy, Angie P. Mejia, Carlos Cardenas-Iniguez, Scott Marek, Anita C. Randolph

**Affiliations:** ^1^Masonic Institute for the Developing Brain, University of Minnesota, Minneapolis, MN, United States; ^2^Department of Population and Public Health Sciences, University of Southern California, Los Angeles, CA, United States; ^3^Mallinckrodt Institute of Radiology, Washington University School of Medicine, St. Louis, MO, United States; ^4^Neuroimaging Labs Research Center, Washington University School of Medicine, St. Louis, MO, United States; ^5^Department of Psychiatry, Washington University School of Medicine, St. Louis, MO, United States; ^6^AI Institute for Health, Washington University School of Medicine, St. Louis, MO, United States; ^7^Department of Pediatrics, University of Minnesota, Minneapolis, MN, United States

**Keywords:** antiracism, research, J-DEI, equity, substance use, critical race theory (CRT), racialization, racism

## Abstract

Racism is embedded in the fabric of society at structural, disciplinary, hegemonic, and interpersonal levels, working as a mechanism that drives health disparities. In particular, stigmatized views of substance use get entangled with racialization, serving as a tool to uphold oppressive systems. While national health institutions have made commitments to dismantle these systems in the United States, anti-racism has not been integrated into biomedical research practice. The ways in which substance use researchers use and interpret race data—without engaging in structural racism as a mechanism of health inequity—can only be described as inadequate. Drawing upon concepts from the Public Health Critical Race praxis, QuantCrit, and an anti-racism research framework, we recommend a set of guidelines to help biomedical researchers conceptualize and engage with race more responsibly in substance use research.

## 1 Introduction

Since the 1950s, substance use has been medically defined as a psychopathology (1). While early definitions once categorized addiction as a “sociopathic personality disturbance” (2), distinct substance abuse and substance dependence labels have existed since 1980 with the American Psychiatric Association’s Diagnostic and Statistical Manual of Mental Disorders (DSM-III) (1, 3). Despite the establishment of a biomedical model of substance use, views of substance use as a personal moral failing have persisted in both the public and medical spheres (4). The moral rhetoric against individuals with substance use disorders (SUDs) goes hand in hand with perceptions that substance use warrants punishment; when paired with prejudice and animosity, these beliefs lay the foundation to target groups of people with the selective enforcement of drug laws, as seen with the US War on Drugs (5). Policies that ignore public health recommendations and employ racism—such as Nixon’s refusal to deschedule and decriminalize cannabis in the 1970s—mold “moral failings” into systemic tools of oppression (6). Hence, it is vital to reject unfounded beliefs about individuals with SUDs that get entangled with racism, which must begin with the scientific community.

Unfortunately, biomedical researchers often report race-related findings without critically considering the systems driving their observations (7–11). Social epidemiologist Dr. Nancy Krieger describes this dilemma as a two-edged sword, where the omission of race data from investigations ignores existing injustice, and the mishandling of race data exacerbates further injustice, reinforcing harmful beliefs about marginalized groups (12, 13). Investigators fall short by not incorporating anti-racist, equity-focused frameworks into their work, such as Critical Race Theory (CRT). Originally a framework for legal analysis, CRT underscores the relationship between race, racism, and power and denotes many ways in which oppression manifests across all forms of expression (14), providing language to examine how social, political, and historical forces give race meaning as a construct (see Table 1 for CRT tenets and principles). Frameworks like CRT can be used to recognize structural racism and racialization as the drivers of differential health outcomes, not race. This gets at a fundamental limitation of the biomedical model of substance use: the model minimizes the impact of psychosocial factors on SUDs (17); in turn, this risks positioning race as a biological driver of SUDs.

**Table 1:** Definitions and major tenets/principles of the anti-racist, equity-focused frameworks that influenced this article’s suggested guidelines for biomedical SUR.

Theory + Definition	Tenets/ Principles
**Critical Race Theory (CRT)** An academic and legal framework that denotes that systemic racism is part of all aspects of American society, providing language through which we can examine the ways in which social, political, and historical forces result in race having meaning as a construct (14).	1.Race is socially constructed. 2.Ordinariness: Racism in the US is normal. 3.Interest convergence: advances for people of color only occur when they serve the interests of dominant white groups. 4.Members of minority groups undergo differential racialization. 5.Intersectionality: No individual can be described by membership in a single group.
**Public Health Critical Race Praxis (PHCR)** An iterative, semi-structured research methodology that guides investigators through a systematic process to conduct self-reflexive, race-conscious research into the root cause of health inequities (13). The four focuses of this research methodology include Contemporary Patterns of Racial Relations, Knowledge Production, Conceptualization & Measurement, and Action.	1.Race consciousness 2.Primacy of racialization 3.Race as social construct 4.Ordinariness of racism 5.Structural determinism 6.Social construction of knowledge 7.Critical approaches 8.Intersectionality 9.Disciplinary self-critique 10.Voice
**QuantCrit** An interdisciplinary framework that applies CRT’s principles to challenge assumptions about the neutrality of quantitative data, offering guidelines on its interpretation and use (15).	1.The centrality of racism 2.Numbers are not neutral 3.Categories/groups are neither “natural” nor given: for “race” read “racism.” 4.Voice and insight: data cannot “speak for itself.” 5.Social justice/equity orientation
**Goings’ Anti-racism Research Principles** A framework on conducting anti-racist research that centers race and racism, encouraging discernment in how a researcher’s positionality relates to a research population, and emphasizing the value of lived experience at each stage of the research process (16).	1.Racism is embedded in structures, policies, and procedures that maintain the status quo. 2.Anti-racist research seeks to dismantle racism. 3.Anti-racist research centers BIPOC experiences. 4.A marginalized racial identity often intersects with other marginalized identities. 5.Anti-racist research foregrounds the importance of self-knowledge. 6.Anti-racist researchers practice what they preach. 7.Anti-racist research involves scientific empowerment, not scientific colonization. 8.Anti-racist researchers prioritize community engagement of the target population. 9.Anti-racist research uses team science to benefit from diverse perspectives. 10.Anti-racist research is concerned with sharing findings with those who support and oppose liberation, social justice, and reduced inequity.

Therefore, in order to engage with race data with diligence, substance use researchers must employ anti-racist praxes in their investigations. Given that many research institutions have been created and maintained through white supremacy, anti-racist progress requires transformative action driven by the perspectives and priorities of minoritized communities (18). Drawing on concepts from the Public Health Critical Race praxis (PHCR) (13), QuantCrit (15), and an anti-racism research framework from Goings and colleagues (16), we recommend a set of guidelines to help biomedical researchers conceptualize and engage with race more responsibly in substance use research (SUR).

## 2 Mishandling race in health disparities and substance use research

The use of race as a broad proxy for structural racism can be extremely harmful to minoritized groups, thereby reinforcing racialized stigma and perpetuating biological determinism (7). Whether explicit or implicit, any use of biological determinism has no place in published literature (7). Across biomedical SUR literature, few studies systematically evaluate whether substance use researchers appropriately define race or report race data. In a recent scoping review of studies from 2000 to 2020 involving maternal–infant dyads with Opioid Use Disorder, investigators found that of the 63 identified quantitative studies that used race/ethnicity in their statistical analyses, only 17 mentioned race/ethnicity in their discussion sections (8). None of the included studies defined race as a social construct, leaving the authors to conclude that SUR “can benefit from a reckoning with how poorly race data have been incorporated to date” (8).

While there are limited critical evaluations of anti-racism in SUR, publications in adjacent health sciences fields highlight a concerning pattern. For example, a systematic review of 329 epidemiology studies published in five prominent journals from 1995 to 2018 found that 61% of studies failed to justify using race or ethnicity data (9). In more recent publications from 2020 to 2021, a review of 192 epidemiology studies revealed that only 23% of articles discussed systemic mechanisms that might drive observed racial health disparities (10). Perhaps even more concerning, three of the identified articles considered biological mechanisms as reasons for racial disparities (10). Similar trends are seen across both public health and medical journals; with broad failures to discuss the role of structural racism in driving health disparities, suggesting that these practices are not outliers but instead the norm (11, 19).

## 3 Guidelines on conducting anti-racist substance use research

The direct link between intersectional oppression and racial health inequity has been recently recognized as a public health crisis (20–22). While institutions like the National Institutes of Health have proposed direct commitments to address racial health inequity in research moving forward (19, 23), such commitments have not yet translated into tangible changes in published biomedical research. Anti-racist research frameworks are especially needed for biomedical SUR, where (1) racism is deeply intertwined with the moralization of substance use, and (2) research findings shape public perceptions, institutional norms, federal policies, and biases from medical providers (4, 24). To this end, we outline a foundational set of guidelines on conducting anti-racist research in the following sections, drawing on principles from PHCR (13), QuantCrit (15), and a framework on conducting anti-racist research (16) (see Table 1 for definitions and tenets/ principles of these frameworks).

### 3.1 Study design considerations

**Guideline 1** : *Employ mixed methods study designs to help contextualize and supplement quantitative findings.*

As previously discussed, the biomedical model of substance use disregards the role psychosocial factors play in SUDs (17), omitting the lived experiences of black, indigenous, and other people of color (BIPOC); these experiences are central to understanding the relationship between structural racism and quantitative findings (13, 15, 16). Considering that addiction neuroscience literature often refrains from engaging with social determinants of health relevant to their study samples (25), it is all the more prudent to seek out additional forms of knowledge and forms of knowing (26). This can be accomplished with mixed methods study designs, where quantitative findings can be contextualized using qualitative representations of lived experience (13, 27, 28). Furthermore, qualitative data from mixed-methods studies can provide information that could not otherwise be captured with quantitative methods, like identifying unexplored manifestations of structural racism (22, 29). These methodologies can be strengthened by applying a community-based participatory research (CBPR) paradigm, which integrates community voice, ownership, and decision making at each stage of the research process (30).

As an example of a mixed-methods research project that employs CBPR, the University of Minnesota’s Randolph Lab is conducting an ongoing comprehensive needs assessment on mental health and substance use in Minnesota youth. This initiative—the Minnesota Youth Needs Assessment (MYNA)—draws on a community of practice model to bring together a network of youth and outreach organizations to collaboratively address the incidence of mental health and substance use among youth aged 12–24 (31, 32). Bringing these stakeholders together allowed us to draw on their experiential knowledge to co-design this study, centering the comfort of youth when broaching certain topics, using unbiased language in study materials, and understanding that youth are the experts of their own experiences. These conversations provided social validity for the study prior to data collection, ensuring that the study methods and aims were aligned with the priorities of, and were accepted by, the community (33, 34). The assessment included a mixture of quantitative and qualitative methods, allowing for an eventual triangulation of data elements to represent a comprehensive picture of the youths’ lived experiences. Although our study is ongoing, we aim to use social context to highlight the systematic factors that are driving mental health and substance use concerns in youth.

**Guideline 2** : *Ensure that samples are not only representative of the minoritized populations being investigated, but also large enough to draw valid conclusions concerning these groups.*

A pivotal goal in human neuroscience—including substance use neuroscience—is building models that are reproducible and generalizable; however, this field has historically relied on small convenience samples from populations that are western, educated, industrialized, rich, and democratic (WEIRD) (35). Although there has been a recent emphasis on larger sample sizes for improved reproducibility of brain-behavior linkages (36), larger samples alone do not guarantee generalizability of brain-based models across individuals from diverse backgrounds. While it may be difficult to find representative samples of certain minoritized groups, it would be inappropriate to draw conclusions using non-representative samples (37). However, representative samples do not guarantee the validity of a model stratified by race, as insufficient sample sizes in stratified groups can result in invalid, harmful findings (37–39).

### 3.2 Diverse, interdisciplinary research teams

**Guideline 3** : *Examine one’s own positionality in relation to a target population, recognizing the limitations of one’s perspectives and embracing diverse, equitable collaborations.*

The social distance between a researcher and a research population is a fundamental consideration factor in ensuring an investigation does not perpetuate scientific racism, stemming from limitations in a researcher’s lived experience (13, 16, 40). Dr. Krieger notes that data does not speak for itself, instead, it is “always produced by people, out of what they observe, fail to see, or suppress in the world in which they live” (12). To this end, biases can determine what research questions get asked, how data are collected and analyzed, how findings are interpreted, and what findings are reported (13, 15, 16, 40). Therefore, it is critical that researchers seek out diverse, equitable collaborations with both fellow scholars and with community stakeholders through CBPR to draw on the wisdom of lived experience.

**Guideline 4** : *Because of how embedded racism is in society, anti-racist research requires a breadth of interdisciplinary expertise.*

Similar to how lived experiences provide socio-contextual expertise to anti-racism research, interdisciplinary teams are necessary to develop robust, comprehensive solutions that address structural racism. Anti-racist research benefits from a range of “unique perspectives, expertise, and approaches” that are drawn from various disciplines, improving the impact, novelty, and reach of research publications (16, 41). Because racism and racialization permeate all aspects of society, specialized (e.g., mixed method), interdisciplinary teams are needed to adequately address health inequity (16, 18, 27, 42).

### 3.3 Use and interpretation of race data

**Guideline 5** : *Whenever possible, measure structural racism directly instead of using race as a proxy for racism or racialization. When analyzing race data, racism or racialization should be framed as risk factors for group differences, not race.*

Since researchers operate under the structures and policies of academic institutions, they are subject to systems that uphold structural racism (13, 16, 18). It is clear that biomedical researchers have not done enough to embrace anti-racism in their work and must move beyond solely documenting inequality (8–11, 13, 19). Therefore it is paramount for researchers to acknowledge structural racism as a driver of health disparities by measuring it directly whenever possible (e.g., racialized economic segregation) (43), rather than using race as a proxy for racism or racialization (7, 13, 16, 44). This shift is needed to eliminate any possibility of erroneous interpretation that racial health disparities are a result of inherent differences or deficiencies (7, 15, 44–46). However, there are circumstances where using race data is required or appropriate, including in identifying populations that are at risk for specific racism exposures (13). Therefore, in these scenarios, researchers must frame racism and racialization as risk factors for outcome differences, not race.

## 4 Discussion

As scientists, our paradigms must reflect the consensus that systemic oppression and social conditions are the drivers of health inequity, otherwise scientific racism persists (7). The many factors that contribute to health disparities (e.g., neighborhood disadvantage) often go unmeasured and ignored with typological thinking in epidemiological studies related to substance use (47); letting researchers “off the hook” from identifying the structural and political forces that give rise to racial social stratification (48). Even when studies do consider some social conditions like the impact of social class on health, they still sidestep addressing structural racism by placing class and race as independent and mutually exclusive of one another, rather than acknowledging their intersectional relationship (38, 49). When researchers omit systemic context, BIPOC populations bear the consequences of structural racism, like lower rates of opioid medication receipt following inpatient admissions compared to white populations, and widening overdose rates (50–52). Hence, it is critical to dismantle the systems that sustain racism and racialization through the use of anti-racist, CRT-informed approaches in SUR.

An interconnection between theory and practice toward social justice must be at the forefront of SUR, from project ideation to implementation. Although there are challenges to integrating anti-racist research into practice that require further attention (e.g., securing funding for community engagement, balancing time-intensive methodologies with systemic pressures for frequent publications), it is nonetheless crucial that we redefine institutional norms to disrupt oppressive systems (16, 53). This includes moving beyond the biomedical model of substance use and shifting the focus of health inequity from race to racism (13, 15–17). While this paper aims to help substance use researchers engage with race more responsibly, we are not the first to make such a call to biomedical researchers, nor are the guidelines presented here exhaustive [e.g., see (38) for considerations on how researchers can operationalize race and ethnicity]. However, given the scarcity of publications systematically examining how the field of SUR handles race, it is necessary to have ongoing, evolving discussions about what anti-racist practices look like within SUR, as seen in adjacent fields [see (10, 13, 15, 16, 38, 54)]. In addition to conducting future systematic reviews, the field must also work to place a greater emphasis on the collective uplift of historically marginalized communities, especially concerning the applicability, acceptability, and community usability of research findings. As noted by race, gender, and law scholar Dorothy Roberts, “Race persists neither because it is scientifically valid nor because its invalidity remains to be proven. Race persists because it continues to be politically useful” (55). It is therefore our duty as substance use researchers to integrate anti-racism into our work in efforts to disentangle racialization from the moralization of substance use, thereby disarming structural racism as a tool of oppression.

### Positionality statement

This manuscript was collaboratively written by a group of early-career researchers whose backgrounds span a spectrum of lived experience and expertise. As a collective, our identities—Mexican, Honduran, Latinx, White, Black, and African—shape our perspectives and our work. Our understanding of SUR as a field stems from our multidisciplinary specializations, including substance use neuroscience, cognitive neuroscience, prenatal substance exposure, pediatric neuroimaging, structural and social determinants of health, community engaged research, sociology, and reproductive justice. Through our engagement and interaction with communities, our perspectives are further informed by community voice. Overall, our outlooks represent a collection of cultural knowledge, expertise, anti-racism scholarship, and community engagement.

## Data availability statement

The original contributions presented in the study are included in the article/supplementary material; further inquiries can be directed to the corresponding author.

## Author contributions

JL: Conceptualization, Writing – original draft, Writing – review & editing. DB: Writing – original draft, Writing – review & editing, Conceptualization. AM: Writing – original draft, Writing – review & editing, Conceptualization. CC-I: Writing – original draft, Writing – review & editing, Conceptualization. SM: Writing – original draft, Writing – review & editing. AR: Writing – original draft, Writing – review & editing, Conceptualization, Supervision.
